# Speech recognition tools for veterinary case learning: enhancing veterinary education with smartphone-based transcription and AI Summarization — a comparative study of workflow and usability

**DOI:** 10.3389/fvets.2025.1690085

**Published:** 2025-12-12

**Authors:** Takuya Yogo

**Affiliations:** Department of Veterinary Surgery, Nippon Veterinary and Life Science University, Tokyo, Japan

**Keywords:** veterinary education, transcription, artificial intelligence, multilingual learning, usability, clinical documentation

## Abstract

**Background:**

Accurate documentation of clinical teaching sessions is critical, particularly in multilingual contexts. Recent advances in smartphone-based speech recognition and large language models (LLMs) may enhance transcription accuracy, streamline case summarization, and improve usability. However, their comparative performance in veterinary settings remains underexplored.

**Objectives:**

This study evaluated the quality, usability, and educational value of smartphone-native transcription compared with Whisper-based transcription and AI-assisted summarization in veterinary ophthalmology education.

**Methods:**

Clinical case discussions (*n* = 5) were recorded and transcribed using (1) iPhone-native speech recognition and (2) the Whisper automatic speech recognition system. Transcripts were further processed into SOAP-format summaries with and without LLM-based summarization. Final-year veterinary students (*n* = 4) and clinicians (*n* = 3) evaluated transcripts and summaries using a 5-point Likert scale across readability, accuracy, clinical clarity, and educational utility. Statistical comparisons were performed using Wilcoxon signed-rank tests.

**Results:**

iPhone-native transcription outperformed Whisper in readability, technical accuracy, and clinical flow (*p* < 0.05). AI-assisted SOAP-format summarization improved clarity and perceived learning value but occasionally introduced minor semantic distortions. Clinicians rated AI-enhanced summaries as more concise and educationally useful than raw transcripts. Both students and clinicians reported reduced cognitive load and usability with smartphone-based transcription workflows.

**Conclusion:**

Smartphone-native transcription combined with AI summarization provides a practical and effective workflow for veterinary education. While Whisper offers cross-device flexibility, its current accuracy in multilingual contexts is limited. Integration of smartphone transcription and LLM summarization may improve documentation, comprehension, and student engagement in clinical teaching.

## 1 Introduction

Clinical case conferences are integral to veterinary medical education, fostering clinical reasoning, enhancing diagnostic decision-making, strengthening professional communication skills, and contributing to the development of veterinary professional identity (1). However, the complexity and rapid pace of these interactions can hinder student comprehension, particularly when it comes to understanding the structured clinical reasoning embedded in professional discourse.

While communication barriers in healthcare settings have been extensively documented (2, 3), limited attention has been directed toward enhancing student learning through the use of structured postconference materials.

Recent advances in automated speech recognition (ASR) and large language models (LLMs) present new opportunities for the development of educational tools. Open-source ASR solutions like OpenAI’s Whisper—trained on 680,000 h of multilingual data (4)—have demonstrated promise in medical transcription. However, these solutions require substantial computational resources. Conversely, native device-based solutions enable immediate deployment without the need for additional infrastructure and also preserve privacy through on-device processing.

LLMs have shown a remarkable ability to process medical information and generate structured summaries in standardized formats such as the subjective, objective, assessment, and plan (SOAP) format (5). In a 2024 study, Van Veen et al. (6) demonstrated that adapted LLMs can match or even surpass the performance of medical experts in clinical text summarization, with 45% of model-generated summaries rated as equivalent and 36% as superior to those created by medical professionals. The integration of accurate speech recognition with intelligent summarization opens the door to comprehensive educational tools capable of real-time transcription, translation, and structured representation of clinical case information.

However, systematic evaluations focusing on the use of different ASR approaches in veterinary medical education remain limited, particularly from the perspective of learning outcomes and implementation feasibility (7).

This study aims to address this research gap by developing and evaluating a practical method for summarizing clinical case conferences in the SOAP format using ASR and LLMs, with the goal of enhancing veterinary students’ understanding of disease processes and their clinical decision-making during ophthalmology rotations. The primary objective of the study was to compare the effectiveness of two transcription methods: Apple’s native iOS speech-to-text functionality and OpenAI’s Whisper ASR model. The specific aims were as follows:

a) To evaluate the quality and usability of the generated SOAP-format summaries, as perceived by veterinary students and clinicians;
b) To assess the practical feasibility of each method in terms of processing time and implementation requirements; and
c) To determine the impact of the generated materials on student comprehension and satisfaction.

Given the educational setting and the limited number of available participants, this research was designed as a *pilot feasibility study* conducted under authentic teaching conditions. The intention was to explore the practicality and educational value of integrating ASR-based summarization tools into veterinary clinical training, rather than to achieve statistical generalization.

## 2 Materials and methods

### 2.1 Case selection and audio data collection

Five ophthalmologic cases were randomly selected from first-time patients at the Veterinary Ophthalmology Service, Veterinary Medical Teaching Hospital, Nippon Veterinary and Life Science University (NVLU) in June and July 2025. Postconsultation case discussions were conducted in Japanese and audio-recorded using the built-in Voice Memo app on an iPhone 15 Pro (256 GB, iOS 18.5), with the files being saved in the M4A format. No personally identifiable information was included in the recordings. The Voice Memo app is natively integrated into iOS and does not have a standalone version number. Transcription was performed using the Apple Speech Framework, which employs the on-device Neural Engine–accelerated ASR pipeline.

Five representative ophthalmologic cases were selected to ensure diversity in clinical content while maintaining feasibility within a single clinical rotation schedule. The number of cases was also limited to minimize the burden on participating students during the busy ophthalmology practicum period, ensuring voluntary participation without interfering with their clinical learning objectives.

### 2.2 Transcription procedures

Each audio file was transcribed using two methods:

1. *iPhone-native transcription*: Apple’s on-device speech-to-text functionality converted recordings directly into text.
2. *Whisper transcription*: The audio files were processed using OpenAI’s Whisper ASR model in a local offline environment. The transcription process utilized a custom Python 3.10 script executed within a Conda-managed virtual environment on a Mac mini (Apple M3 chip, 16 GB RAM) running macOS Sequoia 15.5. Version 1.1.1 of the faster-whisper Python package was installed via PyPI, with the library automatically resampling input audio to a 16 kHz mono waveform during preprocessing. The ‘small’ model variant of Whisper was used, as it provides an optimal balance between transcription speed and accuracy for educational recordings.

### 2.3 LLM summarization

Japanese transcripts were converted into SOAP-format summaries using the Google Gemma 3–4B LLM, executed locally through LM Studio (v0.3.16) on the same Mac mini. All processing was performed offline to ensure data privacy. To protect privacy and ensure that each summary was created without reference to any prior summaries, the chat history was cleared before generating each SOAP output, initiating a cold start for every case.

To maintain consistency and minimize operator bias, a standardized Japanese-language prompt—「このテキストをSOAPに分けてまとめてください」 (“Please summarize this text in the SOAP format”)—was used across all cases. The model output followed the conventional SOAP structure used in clinical documentation.

- *Subjective*: subjective information, such as the owner’s chief complaint and observed findings
- *Objective*: objective information, including physical examination results and diagnostic test findings
- *Assessment*: clinical assessment, including evaluation and differential diagnoses
- *Plan*: planned clinical management, diagnostic instructions, and treatment strategy

This structured output format facilitated downstream evaluation by both students and practicing veterinarians, providing a standardized basis for comparison across transcription methods.

### 2.4 User reviews

Fifteen evaluations were conducted using a 9-item rubric on SOAP-format summaries generated via iPhone-native speech recognition. Each of the three evaluators assessed five cases, resulting in 15 evaluation scores.

#### 2.4.1 Evaluation by students

Four final-year veterinary students at NVLU voluntarily participated with informed consent. On July 4, 2025, each student reviewed paired outputs (Whisper versus iPhone) for each case and completed a questionnaire containing seven items (Q1–Q7), each rated on a five-point Likert-scale (1 = strongly disagree and 5 = strongly agree):

1. The text was easy to read.
2. Technical terms were accurately used.
3. The main points were clearly summarized.
4. The case flow was easy to follow.
5. The content was understandable to Japanese speakers and could be comprehended by them.
6. The summary supported learning.
7. I would like to use this tool in other clinical conferences.

#### 2.4.2 Evaluation by clinical veterinarians

SOAP-format summaries were independently evaluated by three veterinarians—two postgraduate residents and one doctoral student—who were actively involved in ophthalmology training under the supervision of the senior faculty ophthalmologist (T. Y.). This composition reflected the available teaching staff within the ophthalmology service, where one board-certified faculty member oversees both clinical practice and resident instruction. Including these postgraduate evaluators ensured expert-informed yet educationally relevant assessments consistent with the teaching hospital environment.

Each evaluator assessed the summaries using a five-point Likert scale across nine items, including the same seven items used in the student evaluation (sentence readability, accuracy of technical terms, clarity of main points, clinical flow, overall comprehension, perceived learning value, and willingness to reuse), plus two additional items specific to clinical relevance: (1) typographical or semantic issues, and (2) suitability for inclusion in clinical records. These additional items were included to capture professional-level quality and practical utility in a clinical context.

#### 2.4.3 Evaluation by international exchange program students of veterinary medicine

Three final-year veterinary students from the Republic of Korea and three from Taiwan, all participating in an international exchange program at NVLU, were recruited to evaluate the translated SOAP summaries.

Two translation workflows were compared:

1. *Direct*—iPhone-native speech recognition → SOAP-format summary using Google Gemma 3–4B → translation into Korean or Traditional Chinese by the same LLM.
2. *Corrected*—iPhone-native speech recognition → SOAP-format summary → manual correction of Japanese text → translation into Korean or Traditional Chinese by the same LLM (Google Gemma 3–4B running locally).

Each student evaluated SOAP summaries of two randomly selected ophthalmology cases translated into their native language by rating the following seven items on a five-point Likert scale (1 = strongly disagree to 5 = strongly agree):

1. The translated text was natural and easy to read.
2. Technical terms were accurately translated.
3. The key points were clearly summarized.
4. The clinical course of the case was easy to follow.
5. The content was understandable even without knowledge of Japanese.
6. I found it helpful for future learning.
7. I would like to use this tool in other conferences as well.

Transcription and summarization support was initially generated using AI tools (ChatGPT 5, OpenAI), and all outputs were verified, interpreted, and refined by the author. No AI tool was used to generate or alter the final scientific conclusions.

### 2.5 Statistical analysis

Quantitative responses were summarized using descriptive statistics (mean, standard deviation, and median). Paired comparisons between the iPhone and Whisper methods used Wilcoxon signed-rank tests, appropriate for ordinal Likert-scale data with nonparametric distribution. Statistical significance was set at *p* < 0.05. All analyses were conducted in Python 3.10 with SciPy and pandas libraries.

Paired Wilcoxon signed-rank tests were performed to compare scores between the direct and corrected workflows for each question item, with effect sizes (*r*) calculated as |*Z*|/√*n*. Multiple comparisons were adjusted using the Holm method. Internal consistency across the seven evaluation items was assessed using Cronbach’s alpha (*α* = 0.92), indicating excellent reliability among student responses.

### 2.6 Ethical considerations

This study involved the evaluation of educational materials from anonymized veterinary case discussions and the collection of anonymous survey responses. No personal data or patient information was recorded. In accordance with NVLU research ethics guidelines, an ethics application was submitted. As the study posed minimal risk and was not expected to disadvantage participants, it was deemed exempt from a formal ethical review.

All participants participated voluntarily and provided informed consent. Both students and veterinarians were informed, verbally and in writing, that the study was being conducted for educational research purposes, and anonymous questionnaires were collected only from individuals who consented to participate.

## 3 Results

### 3.1 Evaluation by students

Twenty paired evaluations were conducted (4 students × 5 cases × 2 methods). The recorded discussions had a median duration of 343 s (mean: 416 s). Whisper required a processing time approximately equal to the length of the audio file, while transcription on the iPhone was completed nearly instantaneously.

#### 3.1.1 Overall user ratings

iPhone-based transcription yielded higher mean scores across all evaluation items. The overall average ratings were 3.56 ± 0.90 for iPhone and 2.96 ± 1.03 for Whisper (Wilcoxon signed-rank test, *p* < 0.001), indicating significantly higher user satisfaction with iPhone-generated content.

#### 3.1.2 Item-by-item analysis

The seven-item evaluation scale demonstrated excellent internal consistency (*Cronbach’s α* = 0.92), supporting the reliability of student responses. Median scores for each item are summarized in Table 1.

**Table 1:** Comparison of student evaluation scores for SOAP-format summaries generated using Whisper and iPhone transcription methods.

Item No.	Evaluation item	Whisper Mean ± SD	iPhone Mean ± SD	Median (W/iP)	Wilcoxon *Z*	*p*-value	Effect size (*r*)
Q1	Sentence readability	2.96 ± 1.03	3.56 ± 0.90	3/4	−2.98	0.003	0.52
Q2	Accuracy of technical terms	1.96 ± 0.97	2.84 ± 0.87	2/3	−3.15	0.002	0.55
Q3	Ease of comprehension of key points	3.64 ± 0.88	4.04 ± 0.73	4/4	−1.56	0.118	0.27
Q4	Clarity of clinical flow	3.24 ± 0.83	4.00 ± 0.76	3/4	−3.09	0.002	0.54
Q5	Overall comprehension	2.60 ± 1.00	3.32 ± 0.87	3/3	−2.76	0.006	0.48
Q6	Perceived learning value	3.00 ± 0.93	3.52 ± 0.83	3/4	−2.54	0.011	0.45
Q7	Willingness to use for other conferences	2.96 ± 0.85	3.36 ± 0.84	3/3	−1.89	0.059	0.33

Student evaluation scores (*n* = 4 final-year students) for SOAP-format summaries generated using Whisper and iPhone transcription. Scores reported as mean ± SD and median values. Wilcoxon signed-rank test with effect sizes (*r*). *p* < 0.05 considered significant. Q1–Q7 are defined in footnotes. The table depicts the mean ± standard deviation, median scores, Wilcoxon signed-rank test results, and effect sizes for each evaluation item.

SD, Standard deviation.

Median: the central value of a dataset.

Wilcoxon signed-rank test: a nonparametric test used to evaluate differences in medians between two paired groups.

Effect size (*r*): a measure of the magnitude of the observed effect; interpreted as small (0.1), medium (0.3), or large (0.5).

Cronbach’s *α* = 0.92, indicating excellent internal consistency across all evaluation items.

Five of the seven questionnaire items exhibited statistically significant differences in favor of iPhone-based summaries:

- Sentence readability (*p* < 0.001)
- Accuracy of technical terms (*p* = 0.016)
- Clarity of clinical flow (*p* = 0.005)
- Comprehensibility with knowledge of Japanese (*p* = 0.009)
- Perceived usefulness for learning (*p* = 0.018)

“Main points were easy to understand” showed no significant difference (*p* = 0.107), while “willingness to use in future conferences” approached significance (*p* = 0.059). Detailed statistics are presented in Table 1.

#### 3.1.3 Objective assessment of student comprehension

Student comprehension was tested using a list of questions covering presenting complaint, affected eye, diagnosis, and treatment plan for each SOAP summary. All participants achieved 100% accuracy, indicating that essential clinical information was successfully preserved through LLM summarization regardless of the transcription method used.

The processing latency could not be quantitatively measured for the iPhone-native transcription, as the transcribed text appeared instantaneously once the recording was completed and the “Show transcription” option was selected. In contrast, Whisper required approximately real-time processing (about 1.0 × the recording duration).

### 3.2 Evaluation by clinical veterinarians

**Figure 1:**
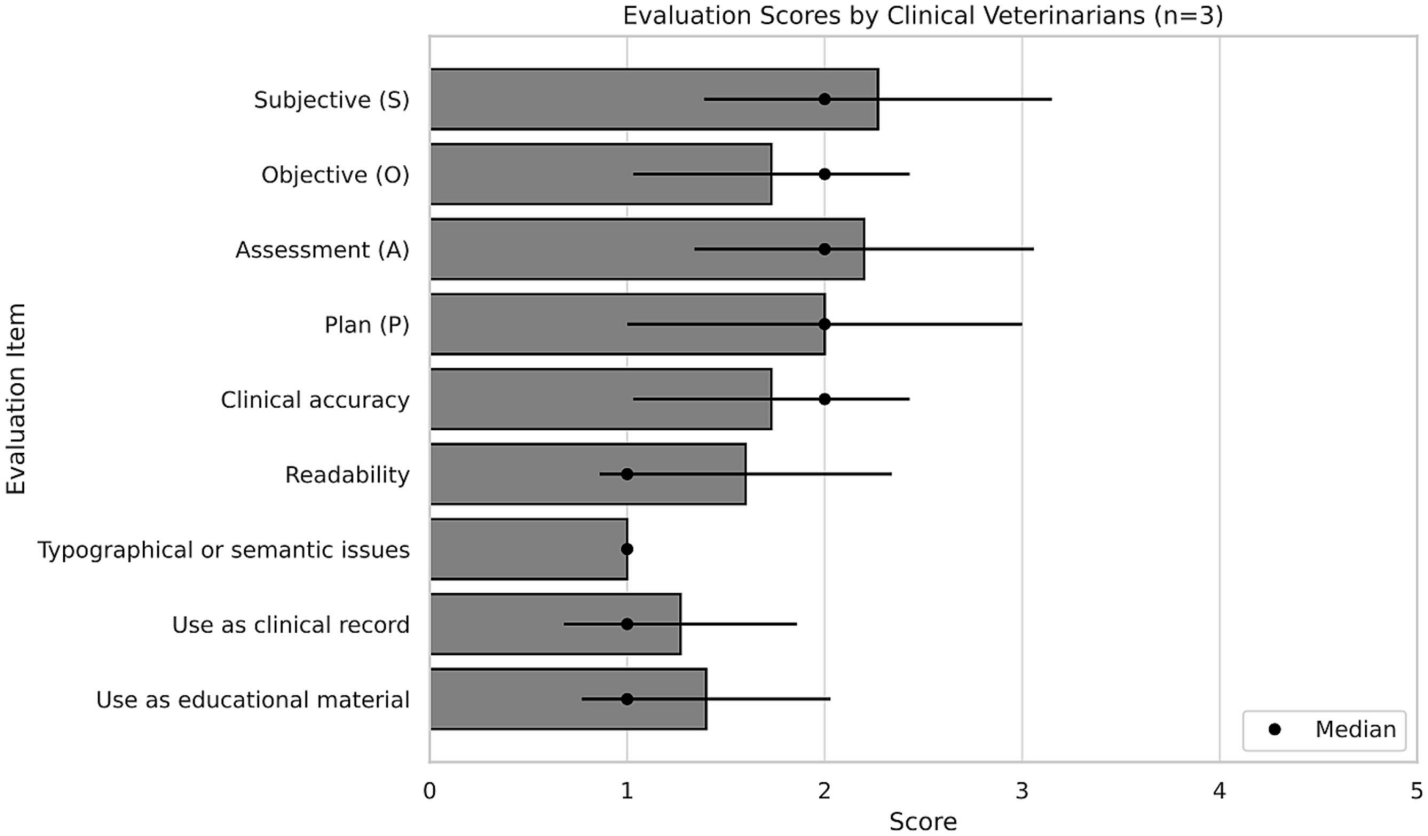
Evaluation scores from experienced clinical veterinarians for SOAP-formatted. Summaries: Bar plot showing mean ± SD scores from three veterinarians (≥5 years’ experience) assessing SOAP-format summaries of five ophthalmology cases. Evaluation domains included: appropriateness of Subjective, Objective, Assessment, Plan sections; clinical accuracy; readability; typographical/semantic issues; and utility for both clinical documentation and education. Scores were rated on a 5-point Likert scale (1 = strongly disagree, 5 = strongly agree), with higher scores indicating more favorable evaluations.

Across 15 evaluations, average scores for each rubric item were generally low. Among them, “Plan” received the highest mean score (2.00 ± 1.00), while “Typographical issues” scored the lowest (1.00 ± 0.00). Median scores clustered around 1–2, indicating limited clinical utility. Notably, ratings for “Suitability for use as clinical records” (1.27 ± 0.59) and “Educational material use” (1.40 ± 0.63) were particularly low, highlighting concerns about the practical applicability of the summaries as shown in Figure 1, Table 2.

**Table 2:** Clinical veterinarian evaluation of SOAP-format summaries generated from iPhone-native transcription.

Item No.	Evaluation item	Mean ± SD	Median	Range
Q1	Subjective (S) appropriateness	1.67 ± 0.82	2	1–3
Q2	Objective (O) appropriateness	1.33 ± 0.52	1	1–2
Q3	Assessment (A) appropriateness	1.73 ± 0.70	2	1–3
Q4	Plan (P) appropriateness	2.00 ± 1.00	2	1–4
Q5	Clinical accuracy	1.60 ± 0.74	1	1–3
Q6	Overall readability	1.87 ± 0.83	2	1–3
Q7	Typographical or semantic issues	1.00 ± 0.00	1	1–1
Q8	Suitability for clinical record use	1.27 ± 0.59	1	1–2
Q9	Usefulness as educational material	1.40 ± 0.63	1	1–2

Clinical veterinarian evaluation (*n* = 3 veterinarians, 15 total evaluations) of SOAP-format summaries generated from iPhone-native transcription. Scores rated on a 5-point Likert scale (1 = strongly disagree, 5 = strongly agree).

*n*: total number of evaluations (evaluators × cases).

Median: the central value of a dataset.

Likert scale: five-point scale ranging from 1 = strongly disagree to 5 = strongly agree.

### 3.3 Evaluation by international exchange program students

No statistically significant differences were observed between the Taiwanese and Korean student subgroups for any of the seven evaluation items (all *p* > 0.05). Across the combined cohort, SOAP summaries in which erroneous Japanese terms—particularly specialized veterinary ophthalmology terminology—had been corrected prior to translation received consistently higher ratings than those translated directly without correction. These improved ratings recorded for the corrected-text workflow were statistically significant overall, with notably higher scores for items Q1 (readability; *p* = 0.002, *r* = 0.74), Q4 (clinical-flow clarity; *p* = 0.003, *r* = 0.71), Q5 (understandable without knowledge of Japanese; *p* = 0.005, *r* = 0.68), Q6 (utility in learning; *p* = 0.004, *r* = 0.69), and Q7 (students’ willingness to use the method in other conferences; *p* = 0.007, *r* = 0.66). These findings suggest that targeted correction of domain-specific terminology prior to translation can substantially enhance the perceived clarity and educational value of the SOAP summary, as well as improve the applicability of this approach to multilingual case summaries, as shown in Table 3.

**Table 3:** Comparison of Direct vs. Corrected summaries in international exchange students’ evaluation.

Item	Description	Median (Direct)	Median (Corrected)	Median Diff	*p*-value	Effect size *r*
Q1	Readability	3	4	1	0.002	0.74
Q2	Technical term accuracy	2	3	1	0.083	0.42
Q3	Key points clarity	3	4	1	0.083	0.42
Q4	Clinical flow clarity	3	4.5	1.5	0.003	0.71
Q5	Understandable without knowledge of Japanese	3.5	4	0.5	0.005	0.68
Q6	Learning usefulness	3	4	1	0.004	0.69
Q7	Willingness to use in other conferences	3	4	1	0.007	0.66

Comparison of student ratings (*n* = 6 final-year students) between Direct and Corrected translation workflows. Results shown as median scores, median differences (Corrected − Direct), and Wilcoxon signed-rank test with effect sizes. *p* < 0.05 considered significant.

Direct: workflow without post-translation correction.

Corrected: workflow revised by faculty after translation.

Median difference: difference in medians (Corrected − Direct).

*p*-value: probability that the observed results occurred by chance; values <0.05 are considered statistically significant.

Effect size (*r*): a measure of the magnitude of the difference; interpreted as small (0.1), medium (0.3), or large (0.5).

## 4 Discussion

This study evaluated two transcription workflows for generating translated summaries of clinical case conferences, aiming to support both Japanese-speaking veterinary students and international students unfamiliar with Japanese. While iPhone transcription yielded significantly higher user satisfaction and operational efficiency, the inclusion of non-Japanese-speaking participants underscores its potential applicability in multilingual veterinary education.

The near-instantaneous turnaround of iPhone transcription enhances educational feasibility by enabling timely feedback and immediate access to transcripts. This real-time, on-device processing requires no technical setup, thereby supporting inclusive learning environments and reducing instructor workload through automation. The exceptional speed of iPhone transcription likely stems from Apple’s on-device speech recognition pipeline, which executes local real-time processing optimized for short recordings. This efficiency likely reflects the hardware acceleration of the Neural Engine integrated into Apple’s A-series chips for iPhone, which enhances speech-to-text conversion through dedicated AI hardware. Cupertino, CA: Apple Inc.; 2025. Available from: https://developer.apple.com/machine-learning/ (accessed 2025 Oct 29). In contrast, Whisper performs sequential decoding of the audio waveform, typically relying on GPU acceleration but also executable on CPU hardware, which results in slower but more flexible processing suitable for longer or more complex recordings.

Beyond technical performance, the integration of AI-based transcription and summarization tools carries important educational implications. First, such tools can reduce instructor workload by automating transcription, translation, and summarization, freeing educators to focus on feedback and individualized guidance. Second, they can support multilingual veterinary education by providing immediate textual outputs accessible to students with varying linguistic backgrounds, thereby enhancing inclusivity and comprehension. Third, structured AI-generated summaries can improve learning efficiency by highlighting clinical reasoning steps and reinforcing SOAP-format understanding. Collectively, these educational dimensions strengthen the relevance and innovation of the proposed workflow, bridging the gap between clinical training and digital learning methodologies in veterinary education.

Five evaluation items significantly favored iPhone transcription—these included readability, technical accuracy, clinical flow, comprehension, and learning value. These results suggest iPhone’s native transcription—potentially owing to integrated language modeling and optimized prosody handling—produces more natural output when processed by summarizing LLMs (8).

In this study, the same LLM, Google Gemma 3–4B, was used for all summarization tasks to ensure that differences in the final SOAP notes could be attributed solely to the transcription process. Gemma 3–4B was selected for all summarization tasks due to three main reasons: (i) the primary research objective was to compare the performance of Whisper and iPhone-native ASR, requiring the LLM component to remain constant; (ii) Gemma 3–4B offers an optimal balance of performance and computational efficiency for local execution on a 16 GB Mac system; and (iii) preliminary evaluations indicated that it outperformed other locally deployable models for this task.

Notably, “main points student comprehension” did not differ significantly between methods, indicating that the LLM extracted content effectively regardless of transcription quality. This suggests that although transcription quality influences readability and engagement, the logical structuring of clinical information remains consistent, provided that prompts are well-designed (9, 10). Although the Gemma 3–4B model demonstrated strong summarization capability, occasional misinterpretations of Japanese ophthalmic terminology in interpreting (e.g., substitution of ‘descemetocele’ with ‘corneal erosion’) were observed. These minor distortions highlight the model’s limited exposure to veterinary-specific corpora and underscore the need for domain-adapted fine-tuning.

Evaluations by clinical veterinarians revealed modest ratings across key dimensions, with median scores of 1–2 on the 5-point scale. While subjective information and assessment categories received acceptable ratings in some cases, objective findings and clinical plans were frequently scored lower. This highlights an important distinction between educational value and clinical reliability: while the summaries effectively promote student understanding, they require refinement to meet clinical documentation standards.

The results indicate limited clinical utility, with particularly low ratings in categories such as readability and suitability for use as formal clinical records. Notably, the ratings for the categories “Objective” and “Clinical accuracy” exhibited greater variability and lower average values, suggesting inconsistencies in capturing precise examination findings and diagnostic coherence. Contrary to expectations, the category “Typographical or semantic issues” received the lowest mean and median scores, reflecting the frequent occurrences of unnatural phrasing or incorrect terminology—particularly for technical vocabulary—an observation consistent with qualitative reviewer comments. These findings highlight the potential of smartphone-based transcription and LLM-based summarization as multilingual educational tools, while emphasizing the need for human supervision when used in professional contexts (11, 12).

In the case of international exchange program students from Korea and Taiwan, correcting the Japanese SOAP text—particularly specialized veterinary ophthalmology terminology—prior to translation consistently improved post-translation comprehension scores. This finding likely sheds light on a limitation of current LLMs, which generally lack robust automated correction capabilities for domain-specific medical terminology (6). Future improvements in LLMs enabling autonomous detection and replacement of inaccurate medical terms could further streamline translation workflows and reduce the need for manual intervention (6, 12). The results of this study also indicate that even relatively small-scale LLMs can produce accurate multilingual translations when the source language is optimized (11). Facilitating the smooth and equitable exchange of accurate medical information across languages is clearly beneficial in the context of global veterinary education (2, 7, 11).

## 5 Conclusion

This study demonstrates the educational utility and operational feasibility of iPhone-native speech recognition combined with LLM summarization for generating multilingual SOAP-format summaries of clinical case discussions. Compared to Whisper-based transcription, the method using iPhone-based transcription achieved significantly higher ratings in readability, clinical flow, technical accuracy, and learning value, while offering near-instantaneous processing.

Evaluations by clinical veterinarians further confirmed the educational utility of this iPhone-based approach while highlighting the need for refinement, particularly in objective clinical data capture and interpretative accuracy. These findings suggest that automated systems may serve as effective educational tools and supplementary resources for instructor support, while requiring human supervision for clinical use.

Evaluations by international exchange program veterinary students from Korea and Taiwan further demonstrated that correcting the Japanese source text, especially specialized veterinary ophthalmology terminology, before translation improved post-translation comprehension. This finding underscores the importance of source-language optimization in maximizing the clarity and educational value of multilingual materials. Thus, the results of this study highlight the potential of automated systems as effective educational tools and supplementary resources for instructor support, while demonstrating that human supervision remains essential for ensuring both linguistic accuracy and clinical applicability.

### 5.1 Limitations

This study has several limitations. First, the number of evaluators (four final-year students and three postgraduate clinicians) was inherently limited by the teaching structure of the ophthalmology rotation. As the only board-certified ophthalmologist on the faculty, the senior author directly supervised all training and evaluation processes. While this restricts statistical generalization, it accurately reflects the authentic composition and workflow of a veterinary teaching hospital.

Second, although evaluations included both Japanese-speaking students and international exchange program students from Korea and Taiwan, the number of evaluators in the latter group was limited and each evaluator reviewed only two cases, which reduces the confidence with which conclusions can be drawn about multilingual applicability.

Third, the quality of output was dependent on the specific LLM used and the design of the prompts, which may affect reproducibility.

Fourth, human supervision remains essential, particularly for cases involving complex clinical reasoning or domain-specific terminology. Finally, transcription and translation accuracy may vary with future updates to the language model or operating system, which could influence the tool’s performance over time.

Nevertheless, these constraints were inherent to the design of this *pilot feasibility study,* which aimed to explore the practicality and educational usability of integrating automatic speech recognition and AI-based summarization into authentic veterinary teaching settings, rather than to achieve statistical generalization. Despite the small scale, the study provides valuable preliminary insights that can guide the design of larger, multicenter investigations. In addition, future multicenter studies involving larger participant groups are planned to validate these findings and assess generalizability across diverse educational contexts.

### 5.2 Future directions

Future research should expand evaluation to larger and more diverse participant groups with varied linguistic backgrounds. Real-time classroom deployment studies could provide insights into the practical implementation of these approaches. Prompt refinement strategies and domain-specific LLM tuning may enhance content fidelity. Exploring applications in interprofessional education and international collaboration could broaden utility in global veterinary and medical education.

## Data Availability

Datasets generated and analyzed during the current study are available from the corresponding author upon reasonable request.
